# Comparison of Low Glycaemic Index and High Glycaemic Index Potatoes in Relation to Satiety: A Single-Blinded, Randomised Crossover Study in Humans

**DOI:** 10.3390/nu10111726

**Published:** 2018-11-10

**Authors:** Sabina S. H. Andersen, Jonas M. F. Heller, Thea Toft Hansen, Anne Raben

**Affiliations:** Department of Nutrition, Exercise and Sports, Faculty of Science, University of Copenhagen, Rolighedsvej 30, DK-1958 Frederiksberg, Denmark; sabinahjorth@hotmail.com (S.S.H.A.); jheller90@hotmail.com (J.M.F.H.); tha@nex.ku.dk (T.T.H.)

**Keywords:** appetite, obesity, eating behaviour, GI, *ad libitum* energy intake, hunger, fullness, prospective food consumption, Carisma, Arizona

## Abstract

High glycaemic index (GI) foods have been proposed to reduce satiety and thus promote overweight and obesity. Generally, potatoes have a high GI, but they also provide many beneficial nutrients and they are a highly important food source globally. In this study, we investigated how a low GI potato affected subjective satiety as compared to a high GI potato. Twenty healthy men (aged 18–40 years; body mass index (BMI) 18–27 kg/m^2^) participated in this single-blinded, controlled, randomised crossover trial. On each of the two trial days, the subjects were given a 500-gram portion of either a low or high GI potato variety (Carisma^®^ low GI and Arizona high GI). Subjective appetite sensations were measured at baseline and at +15 min, +45 min, +75 min, +105 min, and +135 min after consumption of the test meal until an *ad libitum* meal was served at +150 min. No significant differences in the primary endpoint, satiety, were found between the two potato varieties (all *p* > 0.05). Furthermore, no significant differences were found in the secondary endpoints; hunger, fullness, and prospective food consumption, or *ad libitum* energy intake (all *p* > 0.05). In conclusion, the results of this study do not indicate that the GI of potatoes is important for satiety in normal-weight men.

## 1. Introduction

Potatoes are the fourth-most produced food crop worldwide, reaching a total production of approximately 376 million tonnes in 2016 [1,2]. The consumption of potatoes is greatest in the Western world, but potatoes are increasingly becoming a staple food crop in developing countries [3]. The nutrient-to-price ratio of potatoes is overall more favourable than many other fruits and vegetables, which makes the potato an affordable source of beneficial nutrients worldwide [4,5]. Starch is the main macronutrient in potatoes, accounting for 9.1–22.6% of the raw weight depending on variety [6]. In addition to starch, potatoes are an especially a good source of vitamin C, several B vitamins, and potassium. They also contribute to antioxidant activity, particularly if the potatoes are of coloured flesh [5,7]. The skin of the potato is a good source of dietary fibre, and the body of the potato provides resistant starch, which has some of the same beneficial properties for human health as dietary fibre [5]. Lastly, potatoes have high water content (63–87% of the raw weight, depending on variety), giving them a low energy density as compared to other carbohydrate rich foods, such as pasta and rice [6,8]. However, in spite of the clearly beneficial properties of the potato, the effect of the potato on human health has been highly debated in recent years [3,5,7,8,9]. This is due to the fact that many potato varieties, in general, have a high glycaemic index (GI) (GI~77.8) [10]. Consumption of foods with a high GI has been suggested to increase the risk of developing overweight, obesity, type 2 diabetes (T2D), and cardiovascular diseases (CVD) [11,12]. Because of this, some scientists recommend that potatoes should be substituted with low GI sources of starch either from other types of starchy foods or by finding potato varieties with a low GI [3,11,13]. Foods with a high GI have been proposed to increase the risk of overweight and obesity by, among other things, decreasing satiety [11,14].

GI ranks carbohydrates according to their effect on the 2-h postprandial glucose concentrations [15] and is defined as the 2-h area under the curve of the glucose response after ingestion of, usually, 50 grams of available carbohydrates as compared to 50 grams of carbohydrates from a reference (glucose or white bread) [16]. A GI value above 70 is classified as a high GI, a GI value of 56–69 is classified as a medium GI and a GI value of ≤55 is classified as a low GI when using glucose as a reference [17]. To take into account the amount of available carbohydrate consumed in a portion of food, the concept of the glycaemic load (GL) was developed. The GL of a food is defined as the product of the total amount of available carbohydrate in a serving of food and the GI of the food being served [18]. A GL value above 20 is classified as being high, a GL value of 11–19 is classified as being medium and a GL of ≤10 is classified as being low [17]. In general, a standard portion of potatoes has a medium GL (GL~ 17.8) [10]. Foods with a high GI/GL will induce a rapid and high transient increase in the postprandial glucose concentration, while foods with a low GI/GL will induce a lower blood glucose response [11,15]. Thus, foods with a high GI/GL will stimulate a proportionally high insulin response, which might induce a relatively low blood glucose and fatty acid concentration somewhat early in the postprandial period, mimicking a low fuel state [14]. It is this low fuel state, which is proposed to cause reduced satiety and increased hunger after the consumption of foods with a high GI/GL [11,14]. However, high insulin and glucose concentrations, as seen immediately after ingestion of foods with a high GI/GL, have also been proposed to increase satiety [19,20,21]. In relation to this, the review by Bornet et al. (2007) and the study by Anderson et al. (2002) indicate that foods with different GI/GL values might simply lead to increased satiety and decreased hunger at different points in time in the postprandial period [22,23].

Looking into studies evaluating the potato in relation to satiety and GI, the results conflict with the above postulate that the consumption of foods with a high GI decrease satiety. In the study by Leeman et al. (2008), it was found that boiled potatoes gave a greater feeling of satiety than French fries when comparing isocaloric amounts. This was in spite of the fact that the GI of the boiled potatoes was significantly higher than the GI of the French fries. Furthermore, satiety rankings were not found to be associated with postprandial blood glucose response [24]. In addition to the above, the study by Geliebter et al. (2013) found that mashed and baked potatoes, as measured from baseline, at key points gave a greater feeling of satiety and satiation than isocaloric amounts of pasta and brown rice. The GI value of the mashed potatoes was significantly higher than for the other three meals and, again, no association between GI and satiety ratings, or, in this case, *ad libitum* energy intake was found [25]. Together, these results indicate that other parameters than GI/GL are important in regard to satiety, and that in the case of the potato GI/GL seems to have little influence on satiety.

The aim of this study was to investigate how a low GI potato affects subjective appetite sensations when compared to a high GI potato, investigating satiety as the primary endpoint. Based on the above, we hypothesized that the low GI and high GI potatoes would not cause any significant differences in subjective appetite sensations and *ad libitum* energy intake. 

## 2. Materials and Methods 

### 2.1. Subjects

Healthy males aged 18–40 years with a body mass index (BMI) between 18 and 27 kg/m^2^ were recruited for the study. Exclusion criteria included: Chronic diseases (diabetes, cardiovascular diseases, or other metabolic diseases that could affect the results), use of daily prescription medicine, use of dietary supplements that could affect appetite (used within the last month up to or during the study), smoking or use of any nicotine product (used daily within the last three months up to or during the study), over 10 h of strenuous physical activity per week, participation in other clinical studies (participation within the last month up to the study or during the study), food allergies, weight change of more than ±3 kg from screening until the trial was completed, and inability to comply with the procedures required by the study (including an 8–10 h fast before the trial days). The subjects were pre-screened according to the inclusion and exclusion criteria through a questionnaire sent by e-mail. The e-mail also included written information about the study and a folder from the Danish National Committee on Health Research Ethics, “Forsøgspersoners rettigheder i et sundhedsvidenskabeligt forskningsprojekt”. If the inclusion criteria were met in the questionnaire, potential subjects were invited to an information meeting where all the study information was given verbally in groups of 1–3. At the information meeting, the subjects received the folder, “Før du beslutter dig”, also from the Danish National Committee on Health Research Ethics. All subjects still willing to participate in the study after going through the study information were given the opportunity to be screened right away after giving their written informed consent. Each subject was then individually assessed on the inclusion and exclusion criteria, which included a measurement of their height (measured on a Hultafors wall-mounted stadiometer with 0.5 cm accuracy, Hultafors Group Danmark A/S, Ballerup, Denmark) and body weight (measured with a Tanita BWB-600 scale to the nearest 0.1 kg, Tanita Corporation of America, Inc., Arlington Heights, IL, USA). All answers and measurements were noted in the case report form (CRF). After inclusion in the study, the subjects were assigned individual subject numbers. The study was registered at clinicaltrials.gov (NCT03512509) and it was conducted in accordance with the Declaration of Helsinki 1975 (revised 1983). Since normal foods were used as test meals, and no biological samples were taken, the local ethical committee decided that ethical approval of the study was not required.

### 2.2. Experimental Design

The study followed a single-blinded randomised crossover design with two experimental conditions and was conducted at the Department of Nutrition, Exercise, and Sports, University of Copenhagen, Denmark. Recruitment began in February 2018 and the study was completed in April 2018. The subjects were randomised to receive isocaloric amounts of either the low GI (Carisma^®^, Emmeloord, Netherlands) or high GI (Arizona, Emmeloord, Netherlands) potato for breakfast on their first trial day. On both trial days, subjects arrived fasted (8–10 h prior) at 08:00 a.m. at the testing facility. Here, body weight was measured, and the subjects were asked predefined control questions to assure that they were still eligible for the study. The questions were as follows: “What did you use for transportation getting here today?”, “Have you done any sports within the past 48 h?”, “Have you had any alcohol within the past 48 hours?”, “Have you changed your lifestyle radically within the past month?”, “Have you taken any medicine or dietary supplements within the past month?” and “Have you fasted 8–10 h before coming here today?”. All answers were registered in the CRF. The subjects were then guided to the testing room where they at time: −5 min, (at 08:55 a.m.) were asked to fill out the first visual analogue scale (VAS) for the assessment of subjective appetite sensations. At time: 0 min (09:00 a.m.) the subjects received their allocated test meal, which they had to consume over the timespan of 15 minutes. At time: +15 min the subjects were asked to fill out the second VAS measurement. After this, the subjects were asked to fill out VAS measurements at the time points: +45 min, +75 min, +105 min, and +135 min. In total, six VAS measurements were made during each trial day. Two and a half hours (time: +150 min) after the serving of the test meal, the subjects received an *ad libitum* meal (pizza), which they were given 30 minutes to eat. Thereafter, the remaining pizza was weighed and energy intake recorded. The time interval of two and a half hours was chosen to make sure that the subjects did not become so hungry that the satiating effect of the test meal disappeared before serving the *ad libitum* meal. During the trial days, the subjects were allowed to use computers, phones, and talk to each other, as long as the activities did not relate to food, talking about the trial, or involve physical activity. Finally, all subjects had to have at least a seven days washout period between the two trial days.

### 2.3. Standardisation

For 48 h up to the trial days, subjects were instructed to avoid vigorous physical activity, large alcohol consumption (more than the servings of alcohol), as well as abnormal food intake and simply follow their normal day-to-day routines. The subjects were instructed to fast for 8–10 h before arriving at the test facility but were allowed to drink water. Furthermore, the subjects were asked to transport themselves to the test facility with the least possible use of physical activity. Slow cycling (maximum 30 min) was allowed, if cycling was their normal way of transportation. All subjects were given a copy of their answers to the control questions on the first trial day and were encouraged to replicate these behaviours prior to the second trial day. 

### 2.4. Test Meals

The potatoes for the trial, their respective GI-values, and the instructions on how to prepare the potatoes were all provided by the firm Agrico (Emmeloord, Netherlands) [26]. The potato cultivar Carisma^®^ was chosen as the low GI potato (GI~53) [26] and the potato cultivar Arizona was chosen as the high GI potato (GI~93) [26]. Agrico had, as part of their own internal work, measured the GI-values of the potatoes at a certified independent laboratory in Singapore [26]. The potato samples for the testing of the GI-values were both prepared in the exact same way and in the same way as the preparation instructions that were provided to us by Agrico [26]. We followed these instructions carefully and therefore deemed the GI-values representative for the test meals served in the present trial. The test meals consisted of 500 g of sliced and cooked potatoes from either the low GI or the high GI potato variety. The potatoes were washed and then sliced on a mandolin iron to slices of 5 mm. thickness. End pieces were discarded for a more homogenous meal. For each subject, ~510 g of the assigned potato variety was prepared. The potatoes were then placed in pots with 3 L of 37 °C warm water per kg of potato and 24 g of table salt per kg of potato. Hereafter, lids were placed on the pots and the heat of the stove was turned on. Once the water reached a clear boil, the lid was removed, and the potatoes were cooked for four minutes. Thereafter, the cooking water was quickly discarded from the pots and the potatoes were weighed on serving plates into portions of 500 ± 1 g topped with 8 g of regular butter (brand: Lurpak^®^ Salted, Arla Foods amba headoffice, Viby J, Denmark). The plates were then covered with lids and served to the subjects shortly after. The subjects also received a glass of water (250 ± 1 g). The subjects were instructed to consume the entire test meal, including drinking all the water. The test meal was to be consumed over the timespan of 15 min so that the last bite was consumed in the last minute. The test meals were isocaloric (~1420 kJ) and they had similar macronutrient content (see Table 1). All food weighing was done with Sartorius BP6100 Basic Plus Balance (*d* = 0.1 g) (Sartorius Corporate, Goettingen, Germany). Before the trial started, samples of both potato varieties were prepared, using the same method as on the trial days, and sent to Eurofins (Eurofins Steins Laboratorium A/S, Vejen, Denmark) [27], an independent laboratory for nutritional analysis (See Table 1). The exact amount of resistant starch in the two potato cultivates was not determined in the nutritional analysis done by Eurofins. It was accounted for as being part of the total amount of available carbohydrates.

### 2.5. Ad Libitum Meal 

The *ad libitum* meal consisted of frozen “Ristorante Pizza Mozzarella” from Dr. Oetker (Dr. Oetker Danmark A/S, Glostrup, Denmark), chosen for its homogeneity. The pizzas were taken out of the freezer 40 minutes prior to serving and baked at 200 °C for 10 min shortly before serving time in a preheated industrial oven (Rational, SelfCookingCenter, 5 Senses). Three pizzas were placed on a prepared tray and weighed. The subjects were then each served a tray with the three pizzas (a total of ~10,500 kJ), scissors, and a glass of water (250 ± 1 g). Scissors were chosen so that subjects had to actively choose to cut and eat a new piece rather than having pre-cut slices ready to eat. The subjects were instructed to drink all of the served water, not leave behind the pizza crust, and eat until pleasantly satiated. After completion of the meal, the trays were brought back to the kitchen, and the remaining pizza on each subject’s tray was weighed and the energy consumed was calculated (Table 2).

### 2.6. Measurement of Subjective Appetite

Subjective satiety, hunger, fullness, and prospective food consumption was measured using an electronic 100-mm VAS. The electronic version of VAS has been validated against the pen and paper version [28]. The program Evascale© [29] was used to create the 100-mm VAS lines and questions. At each end of the 100-mm line, the most negative and positive answer to the given VAS question was anchored [30]. On each trial day, the subjects were assigned their own tablet that was linked to their subject number. Each subject answered a total of six VAS measurements. In each VAS measurement, the four questions were programmed to appear in a random order to ensure that the subjects read and considered the questions each time. The subjects were instructed to read the question thoroughly and answer with their initial feeling. The questions to be answered were: “How satisfied do you feel?” “How hungry do you feel?”, “How full do you feel?” and “How much do think that you could eat?” The respective answers could range from: “I feel completely empty” to “I could not eat another bite”, “I do not feel hungry at all” to “I feel very hungry”, “I do not feel full at all” to “I feel completely full”, and “I cannot eat anything” to “I can eat a lot”.

### 2.7. Randomisation and Blinding

Sub-investigators assigned the subjects to start with one of the two test meals using a random number generator at random.org/integer-sets/. Only the subjects were blinded in this trial, since the sub-investigators prepared the test meals and analysed all of the data. Each potato variety was assigned the letters A (Carisma^®^) and B (Arizona), which was used in the kitchen to ensure that the subjects received the correct test meals. Once the meals were served, the trays were only marked with subject number. The potatoes were indistinguishable in texture, flavour, and appearance once sliced and cooked, so there was no reason for further blinding. 

### 2.8. Statistical Power

Sample size for appetite sensations were based on the study by Flint et al. (2000). According to this study, an effect size of 5% for mean value rankings of the primary endpoint, satiety, and secondary endpoints; hunger, fullness, and prospective food consumption, can be detected when including 18 subjects and using a paired study design (*α* = 0.05, *β* = 0.80) [30]. To reach statistical power of 0.8 in the secondary endpoint, *ad libitum* energy intake, approximately 26 subjects would be needed, depending on effect size [31].

### 2.9. Statistical Analysis 

All statistical analyses were carried out using R version 3.3.2 (R Core Team, 2016, Vienna, Austria) with Rstudio (Version 1.1.442-© 2009–2018 RStudio, Inc., Boston, MA, USA). Initial data cleaning of VAS scores was performed using visual inspection of all individual curves (using so-called spaghettiograms). VAS measurements outside of ±3 SD was discussed and considered for exclusion. Incomplete data was included in all analyses. Baseline characteristics are shown as mean ± standard deviation (SD). Mixed linear ANCOVA models were used to analyse the difference in incremental area under the curve (iAUC), incremental area over the curve (iAOC), as well as repeated measurements of subjective appetite sensations and the *ad libitum* data. iAOC, iAUC, and *ad libitum* models were adjusted for age, BMI, visiting day, and meal order. Models for repeated measurements of appetite sensations included a time-meal interaction and they were adjusted for age, BMI, visiting day, meal order, and fasting value on the given trial day. No adjustment for multiplicity was applied. Statistical significance was declared using a significance level of 0.05.

## 3. Results

### 3.1. Subjects

A total of 54 individuals responded to our recruitment material, of which 34 did not meet the inclusion criteria. The remaining 20 men attended a screening visit and all 20 were assessed as eligible and included in the study. Baseline characteristics of the included subjects can be seen in Table 3.

**Figure 1:**
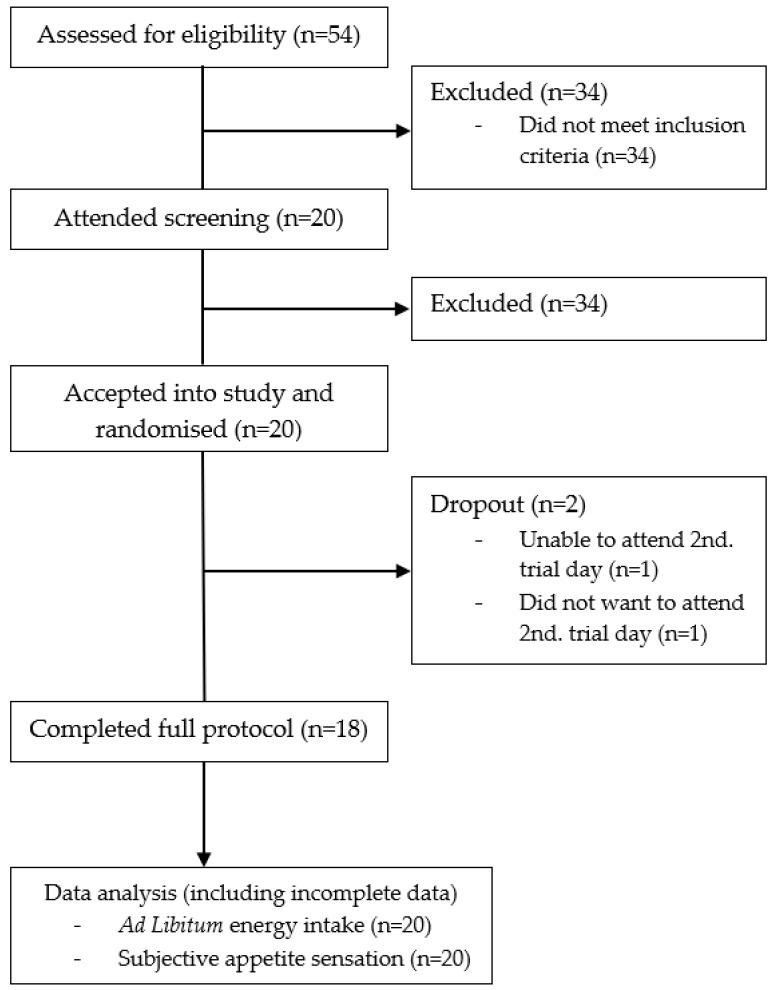
Subject flowchart.

There were two drop-outs. One subject was unable to find time in his schedule to attend the second trial day after completing his first trial day. Another subject was not interested in completing the second trial day for unknown reasons (Figure 1).

### 3.2. Subjective Appetite Sensations

**Figure 2:**
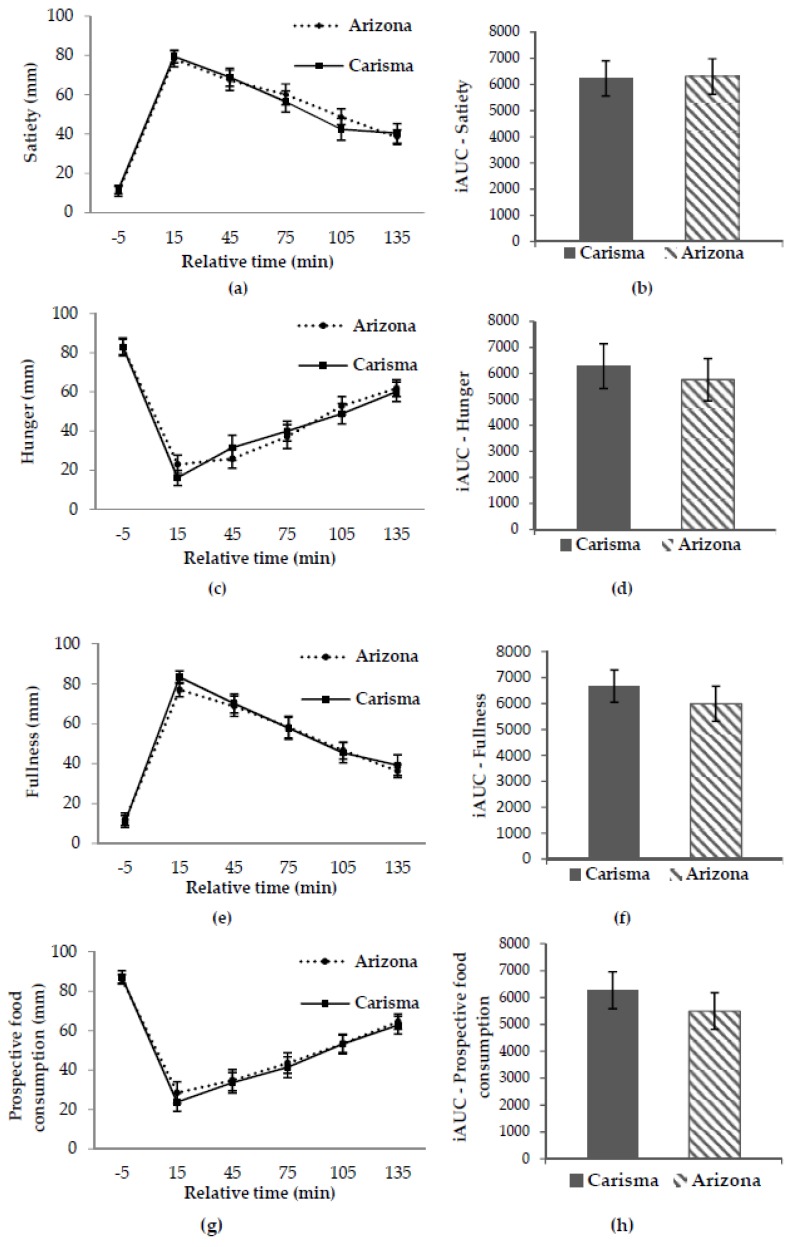
Unadjusted mean curves for satiety (**a**), hunger (**c**), fullness (**e**) and prospective food consumption (**g**) and corresponding incremental areas under/over the curves (iAUC/iAOC) (**b**), (**d**), (**f**) and (**h**). Data are shown as means ± SE. All *p* > 0.05 (adjusted *p*-value). Carisma^®^ (low GI) and Arizona (high GI). SE, standard error; GI, glycaemic index.

The repeated measurement analyses of the primary endpoint, subjective satiety sensation, showed no significant difference in time-meal interaction (*p* = 0.847) or meal effect (*p* = 0.819) between the two potato meals (Figure 2a). Furthermore, no significant difference between the two potato meals was found in the iAUC for subjective satiety (*p* = 0.737) (Figure 2b). 

No significant differences in time-meal interaction or meal effect were found in the secondary endpoints; hunger, fullness, and prospective food consumption, between the two potato meals (Figure 2c,e,g). Furthermore, no significant differences between the two potato meals were found in the iAUC/iAOC for hunger (*p* = 0.213), fullness (*p* = 0.361) and prospective food consumption (*p* = 0.145) (Figure 2d,f,h). 

### 3.3. Ad Libitum Energy Intake

**Figure 3:**
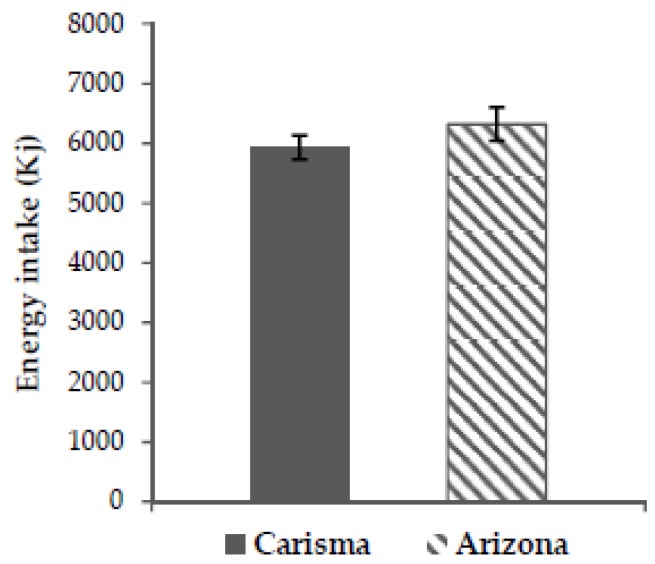
Unadjusted means for *ad libitum* energy intake ± SE. *p* = 0.105 (adjusted *p*-value). Carisma^®^ (low GI) and Arizona (high GI). SE, standard error; GI, glycaemic index.

No significant difference in *ad libitum* energy intake was seen between the two intervention arms (*p* = 0.105) (Figure 3). Adjusted means ± standard error (SE) for Carisma^®^ = 5944.0 ± 255.0 kJ and Arizona = 6321.1 ± 245.3 kJ (Δ 377.1 ± 232.3 kJ). When removing an outliner (difference in *ad libitum* energy intake outside ± 2 standard deviation (SD)), the finding of no difference was further confirmed (*p* = 0.251).

## 4. Discussion

No significant difference in the primary endpoint, subjective satiety, between the two potato varieties in this study was found (all *p* > 0.05). Nor were there any significant differences in the secondary endpoints; hunger, fullness, prospective food consumption, and *ad libitum* energy intake (all *p* > 0.05). This confirms our hypothesis that the low GI and high GI potato would not cause any significant difference in subjective appetite sensations or *ad libitum* energy intake.

Appetite regulation is stimulated by various central and peripheral signals in response to energy balance and the composition of the food ingested [32,33,34,35,36,37,38]. These signals are processed primarily at the level of the brainstem and the hypothalamus, where they stimulate different neurons that either increase or decrease food intake [19]. These central and peripheral signals are however hugely modified by sensory hedonics, sensory stimulation, environmental, and emotional factors [37], thus making appetite regulation a complex matter. To evaluate whether the GI of a certain food is important-as a sole parameter-in relation to subjective satiety, one should therefore control for the following factors; macronutrient composition, energy density, volume, portion size, palatability, and fibre content of the food ingested, while preferably using a crossover design [32,33,34,35,36,37,38]. In our study, we controlled for these parameters by choosing two potato varieties very much alike in nutritional content, texture, flavor, appearance, and by choosing a crossover design. Because we controlled for the above parameters, and because our study found no significant difference in subjective satiety or any of the secondary endpoints, our results do not support the notion that high GI potatoes decrease satiety as compared to low GI potatoes. 

The results of our study are supported by the results of the previously mentioned studies by Leemann et al. (2008) and Geliebter et al. (2013). As mentioned, neither found satiety to be associated with GI when analysing satiety after exposure to different potato meals [24,25], pasta and brown rice [25]. Furthermore, Geliebter et al. (2013) found no association between GI and *ad libitum* energy intake. In a study by Erdmann et al. (2007) the effect of satiating amounts of boiled potatoes, rice and pasta consumed together with 150 grams of pork was analysed for, among other things, satiety rankings, food intake, plasma insulin, and glucose concentrations. The results showed that while boiled potatoes, rice, and pasta resulted in comparable quantities of food intake and satiety rankings, the potato meal resulted in a significantly lower energy intake than the two other meals (potato = 2177 kJ; pasta = 3174 kJ; rice = 2829 kJ). This difference in energy intake did not affect energy intake at the following *ad libitum* meal. Thus, subjects ingested less energy in total when exposed to the potato meal. The glucose response was highest after the ingestion of rice, followed by potatoes and pasta (all non-significant differences), while the potatoes gave a significantly lower rise in insulin than pasta and rice [39]. Similar results were seen in children in a newly published study by Akilen et al. (2016) [40]. Overall, these results might indicate that quantities of food act as a stronger predictor for satiety rankings than the hormonal response. The results of Erdmann et al. (2007) and Akilen et al. (2016) could indicate that the generally low energy density of the potato might be an important satiating property of the potato; one can eat a large volume of potatoes for relatively few calories depending on the preparation style. That energy density is an important factor for satiety was also found in the study by Holt et al. (1995) [34]. They created a satiety index (SI), which was based on ratings of appetite sensations after the ingestion of a 1000 kJ of different types of foods. The authors found that the serving size of the test foods was the strongest predictor of the SI score (GI was not measured). Boiled potatoes produced the highest SI score (323 ± 51) of all the foods included in the study. As many of the other foods included in the study, in general, had a lower GI than boiled potatoes, such as lentils (GI ≈ 35.2), brown rice (GI ≈ 46.5), and brown pasta (GI ≈ 48.3) [10], this again questions the role of the GI of potatoes in relation to satiety. Looking at short-term studies not focusing on potatoes, the findings of no association between GI of a meal and satiety are confirmed in some studies [41,42], while other studies have found an inverse correlation between GI and satiety; low GI meals being more satiating than high GI meals [43,44]. All of the above-mentioned studies were not able to control for the earlier mentioned parameters to the same extent as our study, as they compared different types of foods, meals, and preparation styles. Furthermore, the studies investigated different population groups. To some extent, this might explain the conflicting results seen, but since the potato was not in focus in all studies, the results could also indicate that the role of the GI may vary between different types of foods and food combinations. 

Conflicting results are also observed in long-term studies not focusing on potatoes, as seen in the review by Esfahani et al. (2011). This review included 23 clinical trials comparing a low GI diet with a high GI diet with respect to weight loss. A total of seven out of the 23 clinical trials showed a significantly higher weight loss following the low GI diets as compared to the high GI diets. No significant differences between the remaining 18 trials were found. However, among these 18 trials, 12 favoured the low GI diets and four favoured the high GI diets. In the review, a significant variability between what was considered a low GI and high GI diet was found, and most studies did not match their diets in macronutrients and fibre content [45]. This variability in the use of low GI and high GI diets makes it difficult to compare results between studies, and the lack of standardisation within studies could further indicate that the conflicting results are due to other parameters that are important for satiety. This view is supported by the results of the randomised controlled trials by Sacks et al. (2014), Aston et al. (2007), and Sloth et al. (2004). In these studies, the effect of a low GI diet as compared to a high GI diet was evaluated. Sacks et al. (2014) focused on cardiovascular and diabetic endpoints, Aston et al. (2007) focused on appetite and body weight, while Sloth et al. (2004) focused on both cardiovascular and diabetic endpoints and body weight. The diets in Sacks et al. (2014) were matched for macronutrient composition, fibre, and energy content, while the diets in Aston et al. (2007) and Sloth et al. (2004) were matched for macronutrient composition, fibre content, and energy density in an *ad libitum* setting. Thus, in all three studies, GI was the main difference between the intervention diets. The results of these studies showed no overall difference between most of the endpoints when comparing to the low GI and high GI diets, except for Sloth et al. finding a significant reduction in the concentration of low density lipoproteins (LDL) and Sacks et al. (2014) finding a significant reduction in triglycerides when exposed to the low GI diets compared to the high GI diets [35,46,47]. In a study by Krog-Mikkelsen et al. (2011), a subgroup of the subjects participating in the study by Sloth et al. (2004) was analysed in a single meal study design. They were exposed to a low GI or high GI breakfast meal to evaluate, among other things, subjective appetite sensations and *ad libitum* energy intake after being on the respective low GI and high GI diets for 10 weeks. The two meals differed only in GI, as they were matched in macronutrient composition, energy density, fibre, and energy content. The results of this study showed no overall difference in postprandial sensations of satiety, hunger, and prospective food consumption, but did find a time-meal interaction in ratings of fullness. Fullness being significantly higher (*p* = 0.01) after exposure to the low GI meal. No difference in *ad libitum* energy intake was found between the two groups at lunch [48]. Overall, the results from Krog-Mikkelsen et al. 2011 do not indicate that a high GI diet decreases satiety over time.

Together, all of the above results not only question the role of the GI of potatoes in relation to satiety, but also question the role of the GI in regard to satiety in general.

It is, as mentioned, hypothesized that high GI foods should decrease satiety due to a low fuel state occurring after consumption of high GI foods [11,14]. This hypothesis is questioned in the study by Flint et al. (2006) and the meta-analysis by Flint et al. (2007), both showing that insulin, not glucose, was associated with short term appetite sensations. Thus, higher concentrations of insulin after a meal was associated with decreased hunger and increased satiety [49,50]. Assuming that GI was the only difference between our two study meals due to standardisation, our study does neither support that insulin nor glucose is an important factor in relation to the satiating effects of the potato, as no significant time-meal interaction was found at any time points between the low GI and high GI potatoes. For the same reason, our results do not support the findings that different GI values might give rise to increased satiety and decreased hunger at different points in time in the postprandial period, as stated by Bornet et al. (2007) and Anderson et al. (2002) [22,23]. It should, however, be noted that we did not measure glucose or insulin response after ingestion of the potato meals in our study and thus rely solely on the GI measurements that were provided to us by Agrico [26]. Thus, our results could indicate that other parameters than insulin and glucose might be more important in relation to the satiating effects of the potato. One such parameter could, as mentioned, be the relatively low energy density of the potato. The volume of a meal ingested stimulates satiety through gastric filling and distention, which have been found to be an important mechanism activating satiety signals [36,51]. Another factor, which might make the potato a satiating food, could be its content of the protease inhibitor II (PI2). This inhibitor inhibits trypsin´s proteolytic activity in the small intestine, thus extending the activity of the satiety hormone cholecystokinin (CCK) [52]. However, the effect of PI2 might be masked by other proteins in the potato [8].

The conflicting results within this topic are accompanied by several other points of methodological challenges, which should be considered when evaluating the use of the GI in nutritional recommendations. An important issue to be aware of is the large intraindividual as well as the interindividual variation between GI measurements, as seen in a study by Vega-Lopez et al. (2007) [53]. Another important point to be aware of is the effect of mixed meals on the estimated GI value of a meal. While some results show that the GI of the individual components can be used to predict the GI value of a meal [54], a recent study showed that the calculated GI of a mixed meal was an overestimate when compared to the measured GI value [55]. This might be explained by the fat and protein content of the given mixed meal [56], as both fat and protein are believed to reduce the rate of gastric emptying by stimulating gut hormones, like CCK, and through this effect reduce the glycaemic impact of a meal [36,57]. This is important, as many foods, as the potato, are commonly consumed as part of a mixed meal. It is also important to consider the GL of a food, as this takes into account the standard portion that is normally consumed. In this respect, the potato in general has a medium GL, resulting in a lower glycaemic impact than the high GI of the potato indicates.

When interpreting the results of the present study, one should consider the following. While our study does isolate the effect of the GI quite well, it does not represent at typical mixed meal and the portion size of the potato meals was larger than a normal serving of potatoes [10]. Furthermore, potatoes are not a common breakfast food in Denmark. All of this might have affected the subjective appetite sensations and the *ad libitum* energy intake, thus potentially overshadowing the effect of the GI [58]. It would also have been preferable to know the exact amount of resistant starch in both potato cultivars, as resistant starch is known to affect the GI of a meal and as it has been shown to affect satiety unrelated to the GI [59]. The latter could, again, potentially overshadow the effect of the GI. Furthermore, all subjects were males, which limits the results to this gender. Males were chosen to achieve a homogenous test group and because the menstrual cycle of women can affect appetite sensations and energy intake [60]. Additionally, all subjects were of relatively normal body weight (BMI 18–27 kg/m^2^) and the results could be different in overweight and obese subjects, as the GI might have different effects on individuals with prediabetes and insulin resistance, as seen in the studies by Hjorth et al. (2017) [61] and Hjorth et al. (2017) [62]. Another point to consider is the following: As mentioned, the GI-values that are provided to us by Agrico should represent our test meals quite well due to the standardization of the preparation method. However, it would have strengthened our study if we had measured the glycaemic response ourselves, thus validating the GI-values provided by Agrico. The GI of potatoes, and starchy foods in general, is highly affected by the preparation method, and, as mentioned, the composition of the entire meal being served [5,10,56]. It is essential to be aware of this when using prepublished GI-values in a study design. Thus, scientists should always use representative GI-values, as we did in this study. Another point to consider is that our study only evaluates the short-term effects of the GI of potatoes on satiety, while long-term satiety might be more relevant in relation to the risk of developing overweight and obesity. Lastly, it should be mentioned that decreased satiety is but one hypothesis about how high GI foods might increase the risk of developing overweight and obesity [14,63]. Investigating these are, however, not within the scope of this study, just as the results of this study should not be used to evaluate potatoes’ proposed effect on the risk of developing T2D and CVD due to their, in general, high GI [11,13].

## 5. Conclusions

In conclusion, there was no significant difference in the primary endpoint, subjective satiety, and the secondary endpoints; hunger, fullness, prospective food consumption, and *ad libitum* energy intake between the two potato varieties. This indicates that the GI of potatoes is not in itself a strong predictor for satiety in normal-weight men. 

## Figures and Tables

**Table 1:** Composition of the potato meals.

Nutrients	500 g	500 g	8 g	Total	Total
	Carisma^®^	Arizona	Lurpak^®^	Carisma^®^	Arizona
	Potato (GI~53)	Potato (GI~93)	Butter	Meal	Meal
**Energy**	1175 kJ	1180 kJ	243.0 kJ	1418 kJ	1423 kJ
**Fat**	0 g	0 g	6.6 g	6.6 g	6.6 g
**Carbohydrate**	60 g	60 g	0.1 g	60.1 g	60.2 g
-Total Sugars	4 g	5.5 g	0.1 g	4.1 g	5.6 g
-Fructose	1.55 g	1.35 g	N/A	1.55 g	1.35 g
-Galactose	<0.2 g	<0.2 g	N/A	< 0.2 g	<0.2 g
-Glucose	2.2 g	3.85 g	N/A	2.2 g	3.85 g
-Lactose	<0.2 g	<0.2 g	N/A	< 0.2 g	<0.2 g
-Maltose	<0.2 g	<0.2 g	N/A	< 0.2 g	<0.2 g
-Sucrose	0.4 g	0.35 g	N/A	0.4 g	0.35 g
**Fibre**	7.5 g	8.5 g	0.0 g	7.5 g	8.5 g
**Protein**	5.5 g	5.5 g	0.0 g	5.5 g	5.5 g
**Salt**	2.3 g	2.4 g	0.1 g	2.4 g	2.5 g

Nutritional content of sliced and cooked potatoes analysed after cooling by the independent laboratory Eurofins [27]. Nutritional content of Lurpak^®^ butter (salted) as written on the packaging. GI-values provided by the firm Agrico [26]. GI, glycaemic index; N/A, not available.

**Table 2:** Composition of the *ad libitum* meal.

Nutrients	Pr. 100 g.
**Energy**	1048 kJ
**Fat**	13 g
-Saturated fat	4.4 g
**Carbohydrate**	23 g
-Sugars	2.7 g
**Protein**	10 g

Nutritional content of the pizza as written on the packing (Dr. Oetker).

**Table 3:** Baseline characteristics

Baseline	Mean ± SD	Range
**Age (years)**	25.6 ± 4.3	20–36
**Height (cm)**	183.1 ± 6.0	175.0–198.5
**Weight (kg)**	75.0 ± 9.8	59.2–88.7
**BMI (kg/m^2^)**	22.5 ± 2.3	18.7–26.5

Based on measurements made at screening visits. SD, standard deviation; BMI, body mass index.
